# An integrated, wearable, tubeless micro-negative pressure system for closed incision management: a translational preclinical and clinical study

**DOI:** 10.3389/fmed.2026.1866475

**Published:** 2026-07-13

**Authors:** Yunfan Ti, Jiayang Yue, Xiaohui Cao, Renjie Chen, Fan Wen, Shuo Zhang, Jiangyan Dong, Xiaojiang Yang, Zhanyong Zhu, Zhengfeng Lu, Liang Yuan

**Affiliations:** 1 Department of Orthopedics, Nanjing Jinling Hospital, Affiliated Hospital of Medical School, Nanjing University, Nanjing, China; 2 The First Clinical Medical College of Nanjing University of Chinese Medicine, Nanjing, China; 3 Department of Pathology, Nanjing Jinling Hospital, Affiliated Hospital of Medical School, Nanjing University, Nanjing, China; 4 Nanjing University Institute of Artificial Intelligence Biomedicine, Nanjing, China; 5 Renmin Hospital of Wuhan University, Wuhan, China; 6 Department of Orthopedics, The Second Affiliated Hospital of Soochow University, Suzhou, China; 7 Department of Anesthesiology, Nanjing Jinling Hospital, Affiliated Hospital of Medical School, Nanjing University, Nanjing, China

**Keywords:** closed incision, integrated, microenvironment regulation, micro-negative pressure therapy, translational study, tubeless, wearable

## Abstract

**Background/objectives:**

Closed incision management remains a challenge in postoperative care, largely due to the difficulty of maintaining a stable wound microenvironment in routine clinical practice. Although negative pressure therapy has demonstrated clinical benefits, its real-world effectiveness is often limited by device-related constraints. This study aimed to develop a fully wearable, integrated, tubeless micro-negative pressure system and evaluate its therapeutic potential.

**Methods:**

The system was designed and evaluated in rabbit closed-incision models. Wound exudate control, bacterial burden, inflammatory response, and tissue repair were systematically assessed. In addition, a preliminary clinical study was conducted in patients undergoing minimally invasive spinal surgery to evaluate feasibility, safety, and postoperative performance.

**Results:**

The device significantly reduced exudate accumulation and bacterial burden, and promoted a shift toward a pro-regenerative inflammatory profile. Enhanced tissue repair was observed, including increased angiogenesis, cell proliferation, and collagen deposition. These beneficial effects were consistent across experimental models. In clinical application, the system demonstrated effective exudate management, good usability, and favorable postoperative performance.

**Conclusion:**

This wearable, integrated, tubeless micro-negative pressure system provides an effective and clinically feasible strategy for closed incision management. Its ability to regulate the wound microenvironment and promote tissue repair highlights its translational potential as an alternative to conventional negative pressure therapy.

## 1 Introduction

Effective postoperative incision management is a cornerstone of modern healthcare, directly impacting patient recovery, complication rates, and healthcare resource utilization. Delayed incision healing, excessive exudate accumulation, and surgical site infection (SSI) remain prevalent challenges after surgery, often resulting in prolonged hospitalization, increased nursing workload, and reduced patient quality of life (1–4). As the leading cause of unplanned reoperations, SSI not only worsens patient prognosis but also imposes substantial personal and socioeconomic burdens. Complications such as infection, dehiscence, and delayed healing may extend hospital stays, increase healthcare costs, and in severe cases lead to systemic infection and organ dysfunction (5–13). With advances in medical technology and increasing emphasis on surgical outcomes, optimizing postoperative incision management to reduce SSI and related complications has become a central focus in surgical research.

Negative pressure wound therapy (NPWT) is a widely used modality for wound management and is commonly applied to open wounds, soft tissue injuries, and SSIs (14–18), typically through vacuum-assisted closure (VAC) devices (19). Closed incision NPWT (ciNPT) is gaining increasing popularity for the management and promotion of healing in closed surgical incisions (20–23). By reducing incision tension, optimizing the local microenvironment, and minimizing hematoma and seroma formation, this technique creates favorable conditions for tissue repair, thereby lowering the risks of infection and dehiscence (24–27).

However, conventional NPWT systems are often bulky, noisy, and dependent on external tubing and canisters, which restrict patient mobility and interfere with postoperative activities and rest. These limitations mainly arise from the use of vacuum pumps, one-way valves, and multiple interface components required for pressure generation and monitoring, which increase device complexity, size, and potential leakage points. In addition, air leakage may necessitate frequent pressure compensation, further increasing energy consumption and battery demand. Although several ciNPT-specific devices have been developed to improve portability, many still rely on multi-component assemblies and multiple interfaces, increasing the risk of air leakage and adding complexity to postoperative care. These limitations can compromise patient adherence, increase nursing burden, and reduce the overall efficiency of postoperative incision management in real-world healthcare settings (15, 28).

In this context, focusing on clinically relevant outcomes—including incision healing progression, exudate control, inflammatory regulation, and usability—our study explores the potential of a wearable micro-negative pressure device (WMPD) as a practical, patient-centered solution for closed incision management. Our findings provide evidence supporting the integration of wearable ciNPT technology into routine clinical practice, with the aim of enhancing postoperative recovery and optimizing healthcare delivery.

## 2 Materials and methods

### 2.1 Closed incision creation and grouping

Male New Zealand White rabbits aged 6–7 months were purchased from the Laifu Farm, Pukou District, Nanjing City. All animals were single-housed at room temperature. Before surgery, the dorsum of the rabbits was shaved with Veet. The rabbits were placed in the prone position after anaesthesia with Zoletil 50 (50 mg/mL, 3 mg/kg). The surgical sites were marked with 2-cm lines on the dorsal skin using a ruler, and then made an incision down to the fascia layer with a scalpel. To generate an exudative closed incision model, lipopolysaccharide (LPS) was injected into the incision site. Subsequently, ordinary sterile dressings (OSD), vacuum-assisted closure (VAC) and Wearable Micropressure Device (WMPD) were applied to the wounds. Rabbits with ordinary closed incision (OCI), and highly exudative closed incision (HCI) were divided into six groups with three rabbits in each group. The groups were as follows: OCI + OSD, OCI + VAC, OCI + WMPD, HCI + OSD, HCI + VAC, HCI + WMPD.

### 2.2 Institutional review board statement

The animal study protocol was approved by the Ethics Committee of Nanjing Jinling Hospital (Ethics No.: 2023JLHGZRDWLS-00018). The clinical trial was conducted in accordance with the Declaration of Helsinki and approved by the Ethics Committee of The Second Affiliated Hospital of Soochow University (Protocol Code: LKQ2023045; Approval Date: May 11, 2023). Written informed consent was obtained from the patient for the publication of the clinical images included in this study.

### 2.3 Bacteria counting of wounds

After removing the dressings, the incision surface was cleaned with a sterile saline solution. A predefined tissue sample (~100 mg) was collected from the incision site under aseptic conditions using a sterile scalpel. The tissue was weighed, homogenized in sterile saline, and serially diluted. The diluted samples were separately inoculated onto agar media and incubated at 37 °C in an environment containing 5% carbon dioxide (CO₂) for 48 h under aerobic conditions. Colony-forming units (CFU) were counted and normalized to tissue weight, expressed as CFU/g tissue.

### 2.4 Closed incision assessment: exudate volume

Exudate volume was graded using a subjective assessment method: Grade 0 indicated no exudate, characterized by an obviously dry incision; Grade 1 represented minimal exudate, with the incision remaining moist but no exudate oozing out upon pressure; Grade 2 denoted moderate exudate, where the incision was moist and a small amount of exudate oozed out when pressed; Grade 3 signified copious exudate, with visible exudate retained on the incision and a large amount of exudate flowing out even with slight pressure.

### 2.5 Western blot analysis

Western blot analysis was performed to detect the protein expression levels. In brief, incision tissues were rapidly chilled on ice for the protein extraction via lysis using RIPA buffer (beyotime, China). Following centrifugation, the liquid portion was collected, and the protein concentration in the resulting supernatant was assessed using a BCA kit (beyotime, China). The protein was loaded on BeyoGel™ SDS-PAGE (beyotime, China). Next, the separated proteins were transferred onto the PVDF membranes (Merck, Germany), blocked with 5% skim milk for 1 h. Anti-CD31 (1:1000 dilution ratio, ABclonal, America, A2104), anti-FGF2 (1:1000 dilution ratio, ABclonal, America, A0235), anti-VEGFC (1:1000 dilution ratio, ABclonal, America, A2556), and anti-GAPDH (1:3000 dilution ratio, ABclonal, America, AC033) primary antibodies were incubated at 4 °C overnight. Afterward, the membranes were incubated with HRP-conjugated Goat anti-Rabbit IgG (H + L) (1:10000 dilution ratio, ABclonal, America, AS003), at room temperature for 1 h. Finally, proteins were observed using an enhanced ECL chemiluminescence detection kit (Tanon, China). The protein bands were analyzed with Image J software.

### 2.6 Quantitative RT-PCR (RT-qPCR)

RNA Isolation and Quantitative Real-Time (qRT)-PCR Total RNA was extracted from the incision tissues with the use of TRIzol reagent according to the manufacturer’s instructions (Vazyme, China). cDNA was synthesized using the Hifair® AdvanceFast 1st Strand cDNA Synthesis Kit (Yeasen, China). qPCR was performed with the use of a fluorescence quantitative PCR instrument (Lepu, China) and Hieff UNICON® Universal Blue qPCR SYBR Green Master Mix (Yeasen, China). The oligonucleotide sequences of the primer sets used were as follows: IL-1β (sense, ACC AAC AAG TGG TGT TCT CC; antisense, TCT TTG GGT AAC GGT TGG GG), IL-10 (sense, TCA CCG ATT TCT CCC CTG TG; antisense, GAA GAT GTC AAA CTC ACT CAT GC), TGFβ (sense, ACA GCA TGA ACC GAC CCT TC; antisense, GGT CCT TGC GGA AGT CAA TG), TNFα (sense, TGT CTT CAC CCC CTC TCG TC; antisense, AGG AGG GTG CTC ACT AGA CC), GAPDH (sense, TTT GTG ATG GGC GTG AAC C; antisense, CCC TCC ACA ATG CCG AAG T).

### 2.7 Hematoxylin–eosin (H&E) staining

For histochemical analyses, incision tissues were harvested and divided into two parts: one half was fixed in 4% paraformaldehyde for 3 days, the other half was snap-frozen in liquid nitrogen and then stored at −80 °C for Western blot analysis. The fixed tissues were embedded in paraffin and cut into 4-μm-thick sections. The sections were mounted on glass slides and subjected to routine H&E staining (beyotime, China). Masson’s trichrome stain for collagen fibres in the incision tissues was detected by performing standard protocol and blindly assessed independently (beyotime, China).

### 2.8 Immunohistochemical (IHC) staining

Immunohistochemical (IHC) staining was performed using a primary antibody against Ki67 (Abcam, China, ab15580), to determine cell proliferation. Endogenous peroxidase was quenched with 3% H₂O₂ for 10 min. Non-specific sites were blocked with 5% goat serum for 30 min, followed by overnight incubation at 4 °C with rabbit anti-Ki67. Sections were washed, incubated with HRP-conjugated secondary antibody for 45 min, developed with DAB, counterstained with hematoxylin, dehydrated, cleared and mounted for microscopic evaluation of proliferation indices.

Vasculature in the tissues was assessed by staining for Factor VIII. Sections were deparaffinized, treated with 20 mg/mL proteinase K for 15 min, endogenous peroxidase was blocked for 5 min; sections were washed with PBS and treated with blocking reagent. Factor VIII was applied to the sections and incubated at room temperature for 1 h, rinsed again with PBS repeatedly, and then the sections were incubated with fluoresceine isothiocyanate (FITC)- or rhodamine-conjugated secondary antibody for 30 min. Antibodies were visualized by treating with avidin-biotinylated enzyme complex, then with peroxidase substrate solution for 2 min. They were counter-stained with Mayer’s hematoxylin for 5 min. The stained blood microvessels on each slide were counted in the most vascular area of the section. The integrated option density (IOD) of the digital images was evaluated using Image-Pro Plus software 5.1.

### 2.9 Clinical study design and participants

This was a prospective preliminary clinical feasibility study involving patients undergoing mini-open transforaminal lumbar interbody fusion (MO-TLIF). Patients with closed postoperative lumbar incisions were enrolled after obtaining informed consent. The control group received OSD, while the intervention group received WMPD treatment. Patients with severe systemic infection, coagulation disorders, uncontrolled diabetes, immunodeficiency, or incomplete follow-up were excluded. Postoperative incision healing, exudate management, dressing stability, and device usability were evaluated over a 5-day postoperative observation period. No device-related serious adverse events, skin necrosis, or treatment discontinuation occurred during follow-up.

### 2.10 Statistical analysis

Analyses were conducted using Prism 6 (GraphPad). Differences between groups were assessed using two-way analysis of variance (two-way ANOVA), followed by the Bonferroni *post-hoc* multiple comparisons test. A *p*-value <0.05 was considered statistically significant.

## 3 Results

### 3.1 Morphology and mechanical properties of WMPD

**Figure 1:**
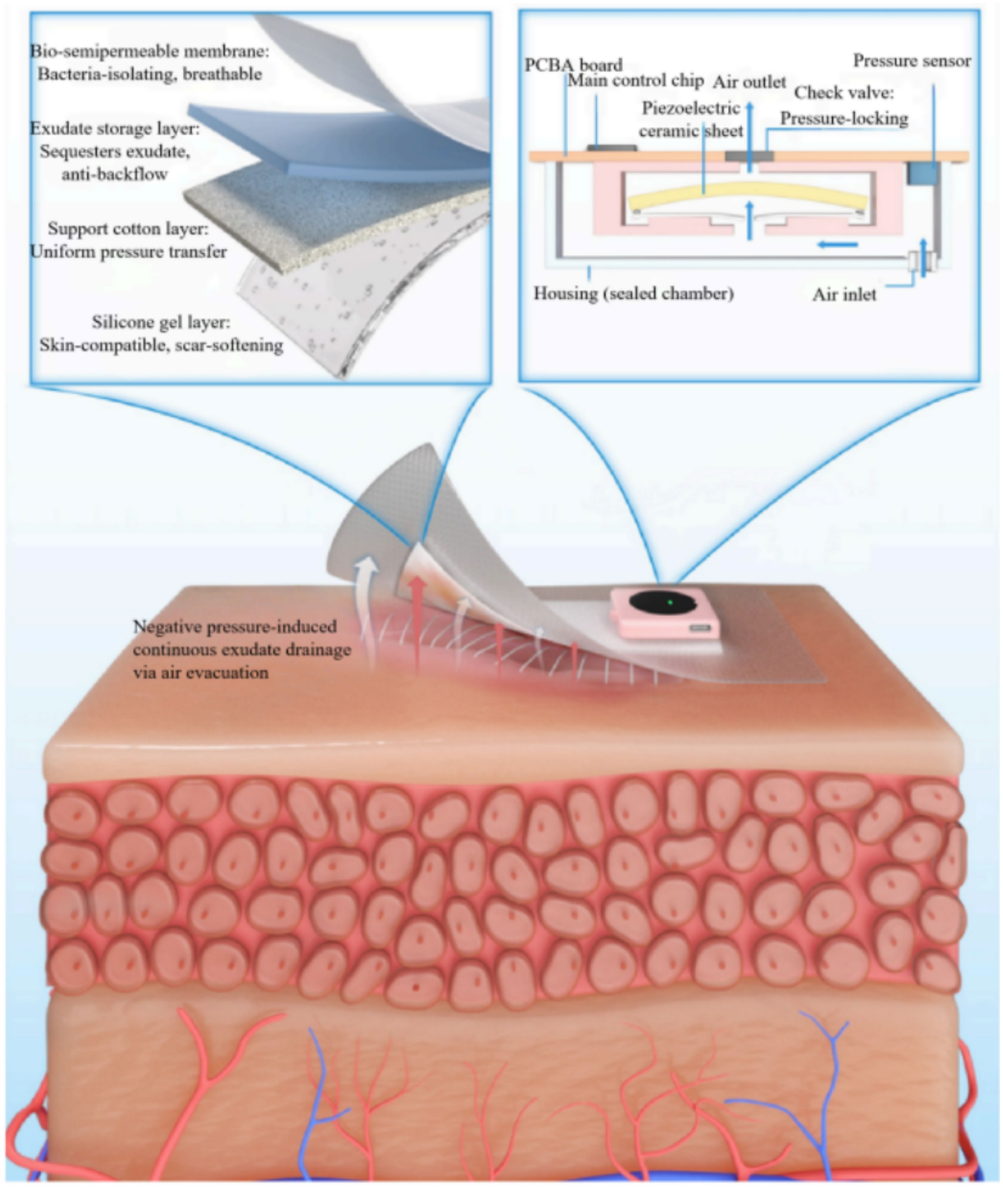
Schematic diagram of the structure and working principle of the WMPD. Upper-left: Layered structure of the wound dressing, consisting of a biological semipermeable membrane, liquid absorption-storage layer, supporting cotton layer, and silicone gel layer. Upper-right: Schematic illustration of the piezoelectric pump module. Piezoelectric ceramic vibration drives unidirectional airflow through the coordinated action of the resonant membrane and check valve, generating negative pressure for wound drainage. Lower panel: *In vivo* application of the WMPD on a skin wound model, where continuous air evacuation establishes a localized vacuum microenvironment for negative pressure wound therapy.

WMPD adopts a laminated structural design in which a valve-like resonant membrane and a piezoelectric diaphragm are vertically stacked, integrating the piezoelectric pump and one-way valve into a single compact unit (Figure 1). This integrated architecture eliminates redundant connectors and reduces dead space commonly observed in conventional vacuum pump systems, resulting in an ultra-compact device with dimensions of 40 mm × 38 mm × 8 mm and a total weight below 16 g, thereby improving portability and wearability.

In terms of mechanical performance, the WMPD demonstrated stable gas output under a pressure load of 26 kPa. When connected to a 100 cc dressing cavity, the device increased the relative pressure from 0 to 100 mmHg within approximately 10 s, indicating rapid establishment of a stable negative-pressure environment. The device operated at a resonant frequency of approximately 24 kHz, which is beyond the human audible range, and generated a noise level below 40 dB during operation. In addition, the piezoelectric actuation mechanism provided high energy-conversion efficiency under low driving voltage conditions, with total power consumption below 1 W. To further reduce standby energy loss, each circuit module was equipped with an independent switching circuit. Owing to the integrated pump-valve structure, the number of connection interfaces was minimized, thereby reducing the risk of air leakage. Furthermore, a closed-loop pressure regulation system, consisting of a pressure sensor and feedback controller, enabled real-time regulation of the piezoelectric pump to maintain pressure stability during operation. The device also supports one-button activation for simplified user operation.

### 3.2 WMPD accelerated closed incision healing in a rabbit model

**Figure 2:**
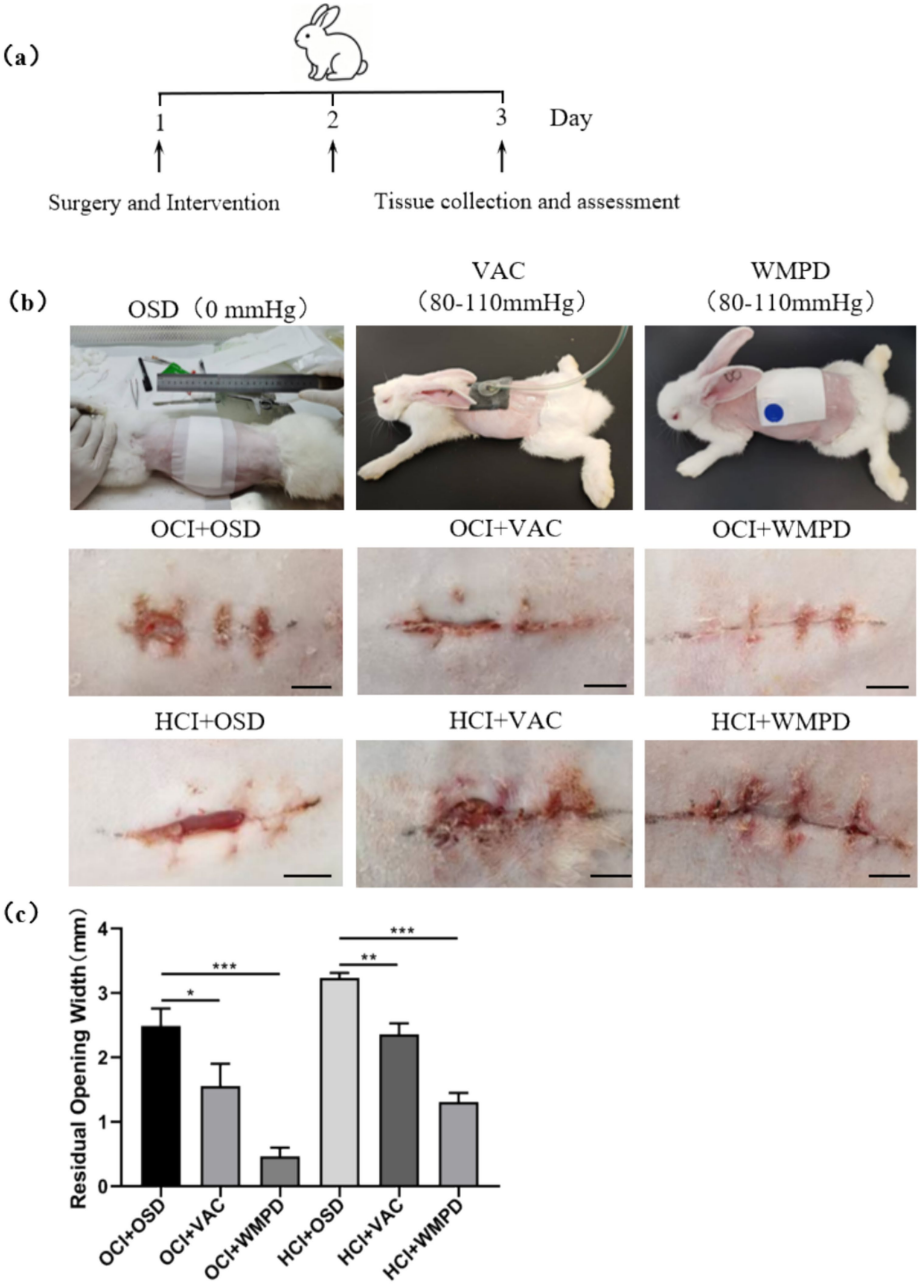
WMPD promotes closed incision healing in rabbit model. **(a)** Experimental timeline for the rabbit closed incision model. Surgery and intervention were initiated on Day 1, with continuous treatment until Day 3, when tissues were harvested for evaluation. **(b)** Phenotypic characteristics of two groups of rabbit closed incision models OCI and HCI after 3 days of treatment with OSD, VAC and WMPD. Scale bar = 0.5 cm. **(c)** The residual opening width of the wounds in each group was quantified on 3 days after treatment.

To investigate the impact of WMPD on the healing of rabbit closed incision, OCI and HCI models were subjected to OSD, VAC, and WMPD interventions, respectively. The results showed that in the OCI model, the residual opening widths of the OSD, VAC, and WMPD groups decreased in turn, and the WMPD group was significantly smaller than the first two groups (Figures 2a,b). Similarly, in the HCI model, the WMPD group also showed the smallest residual opening width, which was significantly better than the VAC and OSD groups (Figure 2c). In conclusion, the WMPD can significantly accelerate incision closure and reduce residual defects after healing, suggesting that it has potential advantages in promoting closed incision healing.

### 3.3 WMPD-mediated ciNPT reduces exudate collection and bacterial colonization

**Figure 3:**
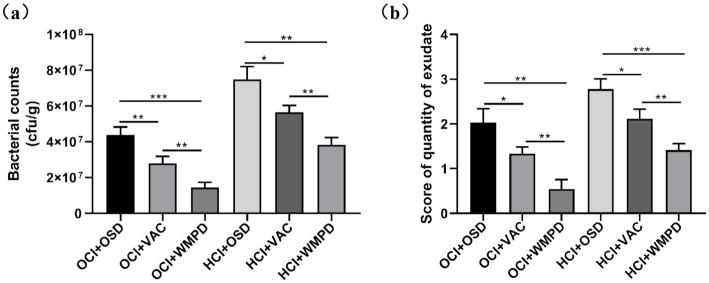
Bacterial load and exudate levels in two incision models across different treatment groups. **(a)** Bacterial counts (CFU/g) at 3 days post-treatment. **(b)** Exudate quantity scores for different treatment groups. Data are presented as the means ± SD, *n =* 3 experiments. ***p < 0.001, **p < 0.01, *p < 0.05, compared with the control.

To quantitatively evaluate incision exudate and bacterial colonization, cumulative exudate volume and bacterial counts in the exudate were measured in each group (Figures 3a,b). In both incision models, the WMPD-treated groups exhibited significantly lower cumulative exudate volumes compared with the OSD and VAC groups (Figure 3a). In addition, bacterial counts in the exudate were significantly reduced in the WMPD groups relative to the OSD and VAC groups (Figure 3b).

### 3.4 WMPD-mediated ciNPT regulates inflammatory response to promote incision repair

**Figure 4:**
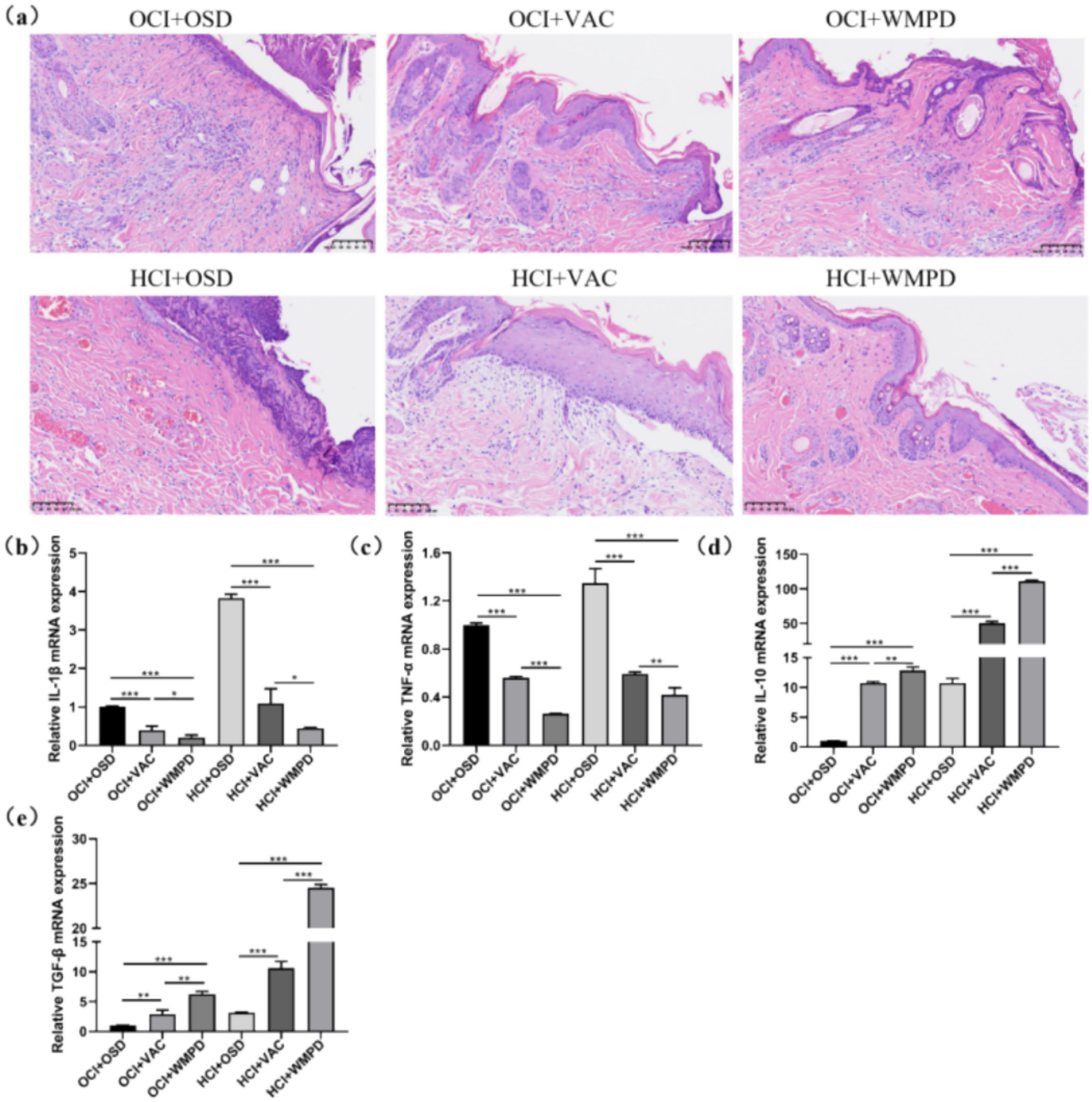
Analysis of hair follicle development and inflammatory response in OCI and HCI models after 3 days of treatment in different groups. **(a)** Representative images of H&E staining of the 3rd day in OCI and HCI wound tissues from different treatment groups. Scale bar = 100 μm. **(b-e)** Statistical analysis of the mRNA expression of **(b)** IL-1β, **(c)** TNF-*α*, **(d)** IL-10, **(e)** TGF-β1 in wound tissue examined by qPCR. Data are presented as the means ± SD, *n =* 3 experiments. ***p < 0.001, **p < 0.01, *p < 0.05, compared with the OSD treatment.

Histopathological analysis was performed using H&E staining (Figure 4a). No hair follicles or sebaceous glands were observed in the OCI + OSD and HCI + OSD groups. In the OCI + VAC and HCI + VAC groups, a small number of hair follicles were detected, with higher counts in the OCI compared to the HCI. In contrast, abundant hair follicles and sebaceous glands were present in the newly formed dermis of both the OCI + WMPD and HCI + WMPD groups, with significantly higher numbers in the OCI + WMPD group compared with the HCI + WMPD group.

Inflammation-related factors in incision tissues were further analyzed. The mRNA expression levels of the pro-inflammatory cytokines IL-1*β* and TNF-*α* were significantly lower in the WMPD-treated groups than in the OSD and VAC groups (Figures 4b,c). Conversely, the expression levels of the anti-inflammatory cytokines IL-10 and TGF-β were significantly higher in the WMPD-treated groups (Figures 4d,e). These expression patterns were consistently observed in both OCI and HCI models.

### 3.5 ciNPT with WMPD exhibits increased angiogenesis

**Figure 5:**
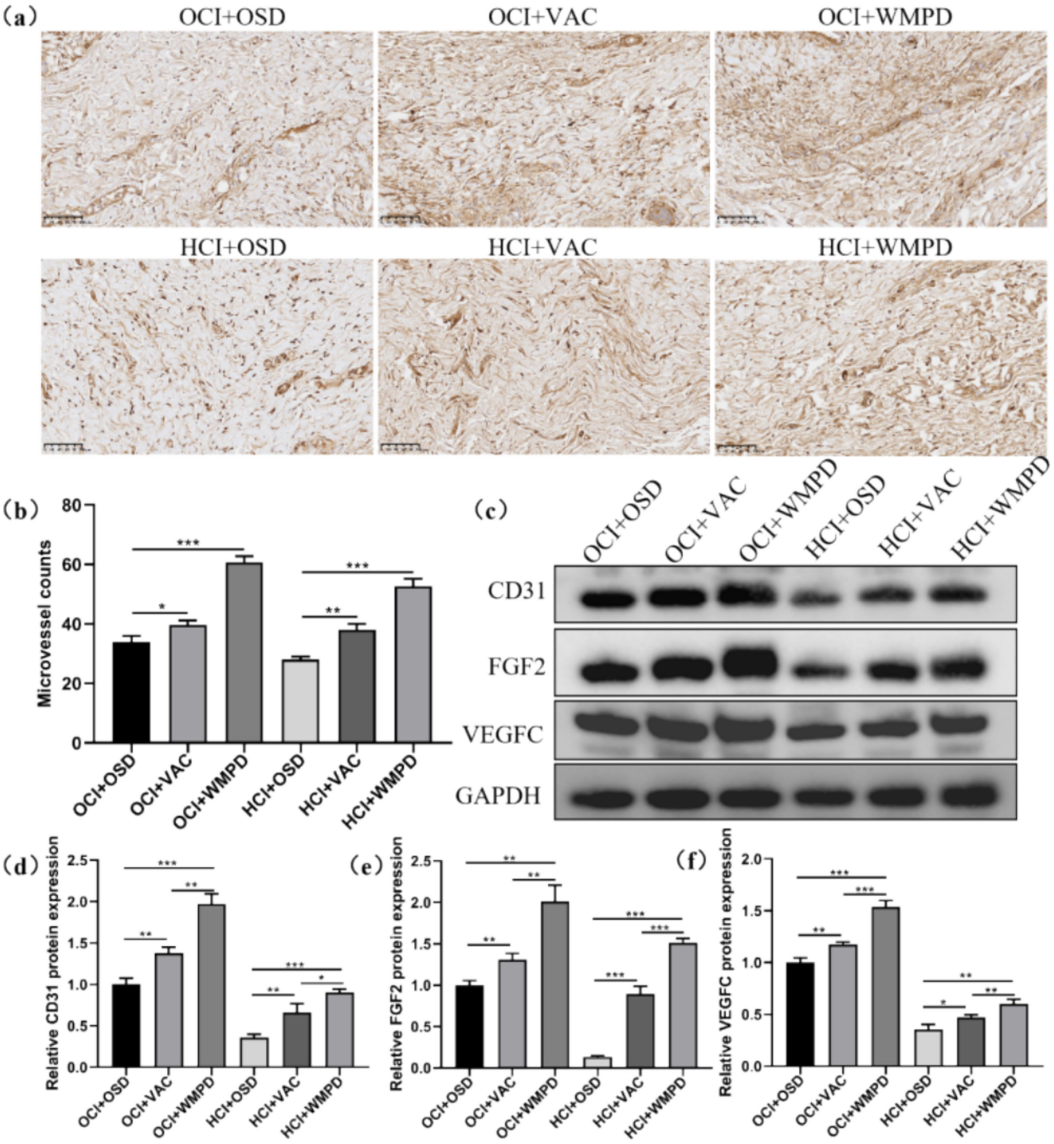
Analysis of microvessel density and expression of key vascular regulatory factors in OCI and HCI models. **(a)** Immunofluorescence staining for Factor VIII on day 3. Microvessels are shown in brown color. Scale bar = 100 μm. **(b)** The count of Microvessels in **(a)**. **(c)** Western blot analysis the expression of CD31, FGF2, and VEGFC on day 3. GAPDH was used as the loading control. **(d-f)** Statistical quantification of the protein expression levels of CD31 **(d)**, FGF2 **(e)**, and VEGFC **(f)** in (**c**). Data are presented as the means ± SD, *n =* 3 experiments. ***p < 0.001, **p < 0.01, *p < 0.05, compared with the control.

To assess angiogenesis in incision tissues, factor VIII was used as a vascular marker for immunohistochemical analysis (Figures 5a,b). In both OCI and HCI models, the WMPD-treated groups (OCI + WMPD and HCI + WMPD) showed more extensive factor VIII–positive staining compared with the OSD and VAC groups (Figure 5a). Quantitative analysis demonstrated that microvessel density was significantly higher in the OCI + WMPD group than in the OCI + OSD and OCI + VAC groups, and similarly increased in the HCI + WMPD group compared with the HCI + OSD and HCI + VAC groups (Figure 5b).

The expression of angiogenesis-related proteins was further analyzed by immunoblotting. The protein levels of CD31, FGF2, and VEGFC were significantly higher in the WMPD-treated groups than in the OSD and VAC groups in both OCI and HCI models (Figures 5c–f).

### 3.6 Effects of WMPD-mediated ciNPT on cell proliferation and collagen

**Figure 6:**
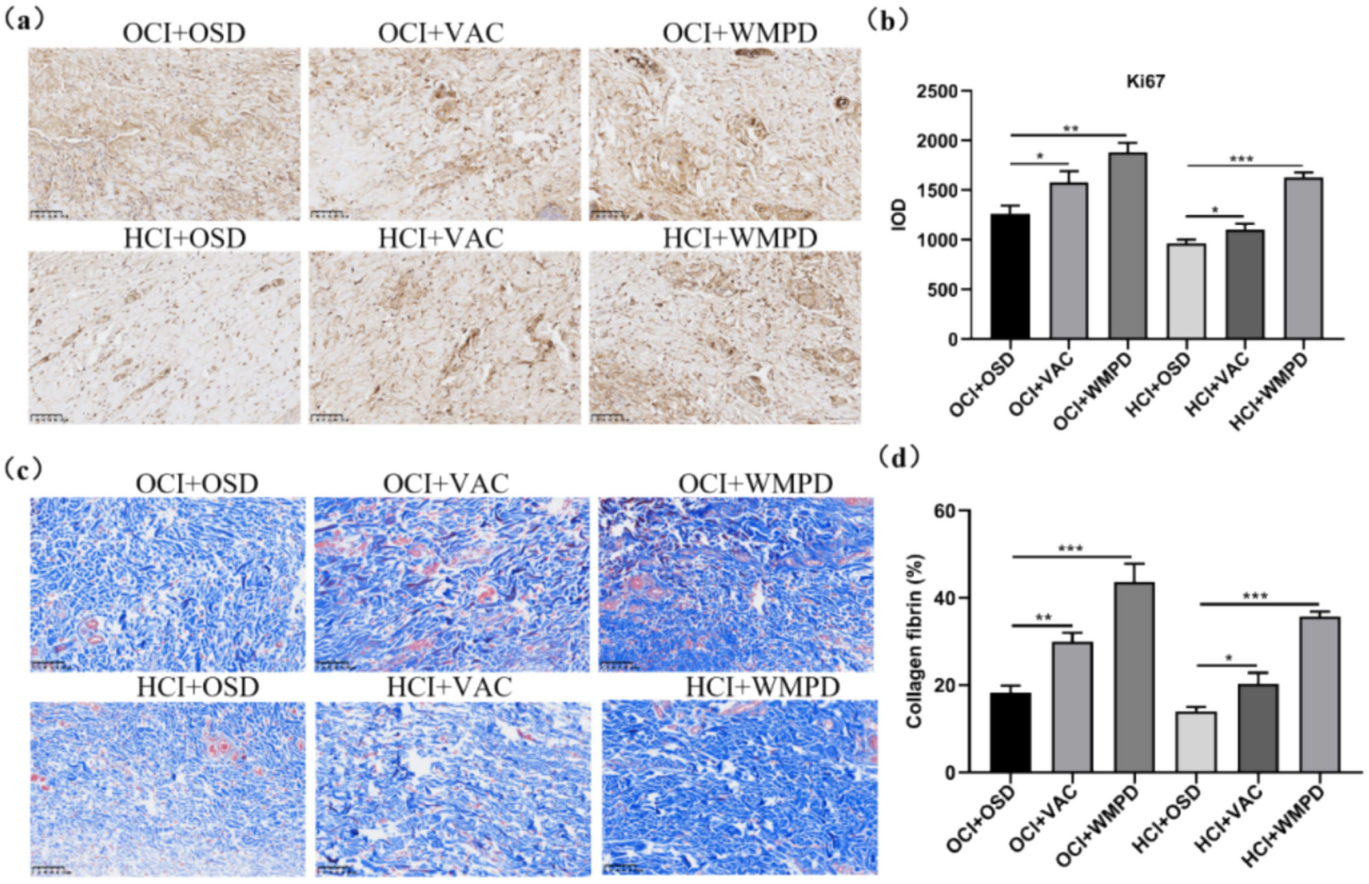
Analysis of cell proliferation and collagen organization in OCI and HCI models treated with OSD, VAC, and WMPD. **(a)** Immunofluorescence staining for Ki67. Scale bar = 100 μm. **(b)** Statistical quantification of the IOD value of Ki67 in **(a)**. **(c)** Masson’s staining histological images. Scale bar = 100 μm. **(d)** Statistical quantification of the Masson’s staining in **(c)**. Data are presented as the means ± SD, *n =* 3 experiments. ***p < 0.001, **p < 0.01, *p < 0.05, compared with the control. IOD, integrated option density.

To evaluate cell proliferation at the incision site, Ki67 was used as a proliferation marker. Immunohistochemical staining and integrated optical density (IOD) analysis showed that, in both OCI and HCI models, the OCI + WMPD group exhibited significantly more Ki67-positive cells than the OCI + OSD and OCI + VAC groups. Similarly, the HCI + WMPD group showed a significantly higher number of Ki67-positive cells compared with the HCI + OSD and HCI + VAC groups (Figures 6a,b).

Collagen deposition in incision tissues was further assessed by Masson’s trichrome staining. The WMPD-treated groups (OCI + WMPD and HCI + WMPD) displayed more abundant and densely distributed collagen fibers than the corresponding OSD and VAC groups (Figure 6c). Semi-quantitative analysis confirmed that the collagen fiber proportion was significantly higher in the OCI + WMPD group than in the OCI + OSD and OCI + VAC groups, and similarly increased in the HCI + WMPD group compared with the HCI + OSD and HCI + VAC groups (Figure 6d).

### 3.7 WMPD-mediated ciNPT in human clinical trials: promotion of closed incision healing progression

**Figure 7:**
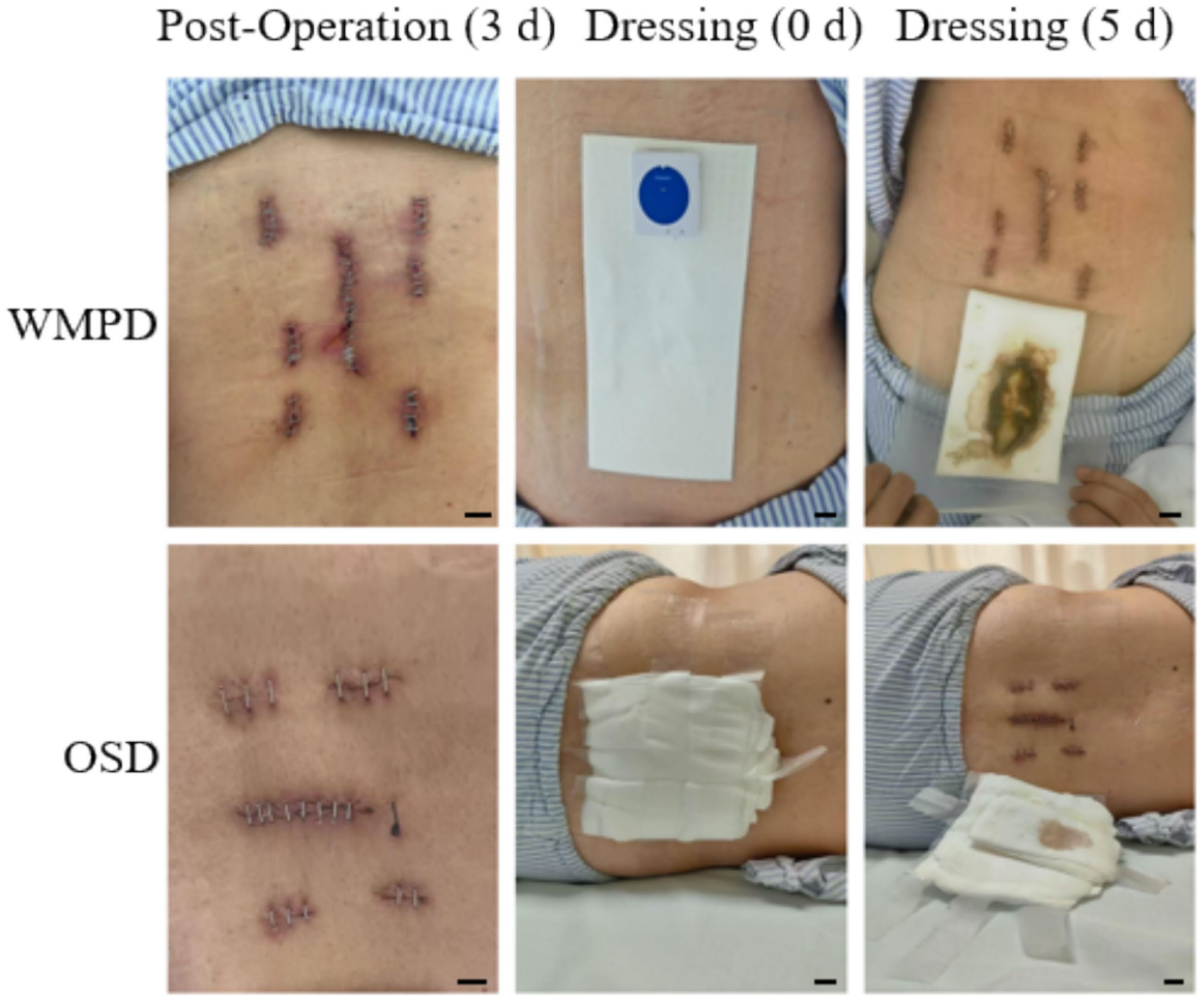
Clinical trial: closed incision healing efficacy of WMPD and OSD in patients after MO-TLIF. The upper row presents the WMPD group, sequentially displaying the wound status at 3 days post-operation, the appearance of the WMPD device *in situ* at 3 days post-operation, and the wound healing outcome after 5 days of dressing application. The lower row shows the corresponding observations for the OSD group. Scale bar = 1 cm.

Finally, a clinical trial was conducted to evaluate the safety and efficacy of WMPD in closed incision treatment. Patients at 3 days after mini-open transforaminal lumbar interbody fusion (MO-TLIF) surgery were enrolled (Figure 7). Although MO-TLIF reduces the need for traditional drainage, postoperative incisions still produce measurable exudate (29).

Postoperative incisions were treated with either WMPD or ordinary sterile dressings (OSD). Compared with OSD, the WMPD group showed a faster incision healing rate. With respect to exudate management, diffuse exudate accumulation was observed in the OSD group, whereas the WMPD group exhibited more concentrated and effective exudate absorption. In addition, dressing displacement occurred more frequently in the OSD group, while WMPD maintained stable adhesion at the incision site throughout the observation period. Overall, these clinical results indicate that WMPD provides improved incision healing and exudate management compared with OSD in patients after MO-TLIF surgery.

## 4 Discussion

Effective management of closed surgical incisions remains a clinical challenge, particularly due to postoperative exudate accumulation, inflammation, and delayed tissue remodeling (10, 12, 13). In this study, we developed and evaluated a wearable micro-negative pressure device (WMPD) and demonstrated its therapeutic benefits in both preclinical models and a clinical setting. Our findings indicate that WMPD promotes closed incision healing through coordinated regulation of exudate control, inflammation, angiogenesis, and tissue remodeling.

Unlike conventional vacuum-assisted closure systems that rely on bulky motors, complex mechanical transmission, and external tubing, WMPD employs a highly integrated piezoelectric pump based on the inverse piezoelectric effect. This design enables continuous and stable directional airflow without a motorized structure, allowing the pump volume to be reduced to <20% of traditional vacuum pumps. As a result, WMPD achieves ultra-quiet operation (< 40 dB), low power consumption (< 1 W), rapid pressure establishment, and stable closed-loop pressure regulation. These technical advantages directly translate into improved wearability and uninterrupted therapy, addressing major limitations that restrict the routine clinical use of conventional ciNPT systems.

Postoperative exudate accumulation is a critical determinant of incision stability and infection risk (30). In both ordinary and highly exudative closed incision models, WMPD significantly reduced cumulative exudate volume and bacterial burden compared with sterile dressings and conventional VAC devices. Continuous micro-negative pressure provided by WMPD facilitates efficient fluid evacuation while maintaining a closed, protected wound environment, thereby limiting bacterial colonization and local tissue maceration. Appropriate modulation of inflammation is essential for successful incision repair (30, 31). WMPD treatment was associated with reduced expression of pro-inflammatory cytokines, including IL-1*β* and TNF-*α*, alongside increased levels of anti-inflammatory mediators such as IL-10 and TGF-β. This balanced inflammatory profile supports the transition from the inflammatory to the proliferative phase of wound healing. Consistently, enhanced angiogenesis, increased cellular proliferation, and greater collagen deposition were observed following WMPD treatment, indicating improved tissue regeneration and structural maturation at the incision site. It should be noted that the mechanistic investigation in this study is limited by the small number of detected biomarkers. To fully uncover the molecular basis of WMPD-mediated wound healing, multi-omics analyses including transcriptomics, proteomics and immune phenotyping will be implemented in future studies.

Importantly, the translational feasibility of WMPD was supported by clinical evaluation in patients undergoing mini-open transforaminal lumbar interbody fusion. Compared with conventional sterile dressings, WMPD demonstrated superior exudate management, improved incision healing, and greater dressing stability. Its ultra-thin, lightweight, and tubeless design enables continuous negative pressure therapy without compromising postoperative mobility or patient comfort, key considerations for real-world clinical adoption. Nevertheless, several limitations of this study should be acknowledged, consistent with the exploratory and preliminary nature of this translational investigation. First, the relatively small clinical cohort may limit the statistical robustness and generalizability of the observed clinical outcomes. Second, this study primarily focused on short-term postoperative healing parameters, and long-term follow-up data are lacking; therefore, the effects of WMPD on scar maturation, chronic wound-related complications, and long-term patient outcomes remain to be further elucidated. Third, clinical validation was conducted only in patients undergoing lumbar interbody fusion surgery, and the applicability of WMPD across other surgical procedures and wound types warrants further investigation. Notably, this study focused on closed-incision wounds, and future work will explore its feasibility in open or more complex wound settings, potentially expanding its clinical applicability.

In summary, WMPD integrates stable micro-negative pressure with a wearable design, providing a compact, low-noise, energy-efficient, and patient-centered ciNPT solution with strong potential for routine postoperative care and broader clinical translation.

## 5 Conclusion

In this study, we report the development and validation of a novel wearable micro-negative pressure device (WMPD) for closed incision negative pressure therapy. In both preclinical models and a clinical study, WMPD outperformed conventional sterile dressings and traditional VAC systems in promoting incision healing. It effectively controlled exudate and bacterial burden, modulated inflammatory responses, enhanced angiogenesis and cell proliferation, and improved collagen deposition—key determinants of optimal wound repair.

The WMPD’s ultra-thin, lightweight, and tubeless design enables continuous, stable micro-negative pressure therapy without restricting patient mobility or comfort, addressing a major limitation of conventional ciNPT devices. Clinical application in patients undergoing mini-open transforaminal lumbar interbody fusion confirmed its safety, ease of use, and translational potential.

Collectively, these findings position WMPD as a clinically adaptable, patient-centered strategy for postoperative incision management. Its combination of therapeutic efficacy, usability, and mobility-friendly design offers a practical and effective alternative to conventional approaches, with the potential to improve outcomes in a range of surgical settings.

## Data Availability

The original contributions presented in the study are included in the article/supplementary material, further inquiries can be directed to the corresponding authors.

## References

[1] Borejsza-Wysocki M Bobkiewicz A Francuzik W Krokowicz L Walczak D Szmeja J . Effect of closed incision negative pressure wound therapy on incidence rate of surgical site infection after stoma reversal: a pilot study. Wideochir Inne Tech Maloinwazyjne. (2021) 16:686–96. doi: 10.5114/wiitm.2021.106426, 34950263 PMC8669980

[2] Javed AA Teinor J Wright M Ding D Burkhart RA Hundt J . Negative pressure wound therapy for surgical-site infections. Ann Surg. (2019) 269:1034–40. doi: 10.1097/SLA.0000000000003056, 31082899

[3] Zukowska A Zukowski M. Surgical site infection in cardiac surgery. J Clin Med. (2022) 11:6991. doi: 10.3390/jcm11236991, 36498567 PMC9738257

[4] Lemaignen A Birgand G Ghodhbane W Alkhoder S Lolom I Belorgey S . Sternal wound infection after cardiac surgery: incidence and risk factors according to clinical presentation. Clin Microbiol Infect. (2015) 21:674.e11–8. doi: 10.1016/j.cmi.2015.03.025, 25882356

[5] Fuglestad MA Tracey EL Leinicke JA. Evidence-based prevention of surgical site infection. Surg Clin North Am. (2021) 101:951–66. doi: 10.1016/j.suc.2021.05.027, 34774274

[6] Leaper DJ Tanner J Kiernan M Assadian O Edmiston CE. Surgical site infection: poor compliance with guidelines and care bundles. Int Wound J. (2015) 12:357–62. doi: 10.1111/iwj.12243, 24612792 PMC7950697

[7] Edmiston C Jr Spencer M Gunja NJ Holy CE Ruppenkamp JW Leaper DJ. Longitudinal rates, risk factors, and costs of superficial and deep incisional surgical-site infection (SSI) after primary and revision total knee arthroplasty: a US retrospective claims database analysis. Infect Control Hosp Epidemiol. (2023) 44:1587–95. doi: 10.1017/ice.2023.10, 36726345

[8] Sullivan SR Fletcher DRD Isom CD Isik FF. True incidence of all complications following immediate and delayed breast reconstruction. Plast Reconstr Surg. (2008) 122:19–28. doi: 10.1097/PRS.0b013e3181774267, 18594356

[9] O'Leary DP Peirce C Anglim B Burton M Concannon E Carter M . Prophylactic negative pressure dressing use in closed laparotomy wounds following abdominal operations: a randomized, controlled, open-label trial: the P.I.C.O. Trial. Ann Surg. (2017) 265:1082–6. doi: 10.1097/SLA.0000000000002098, 27926575

[10] Curran T Alvarez D Del Valle JP Cataldo TE Poylin V Nagle D. Prophylactic closed-incision negative-pressure wound therapy is associated with decreased surgical site infection in high-risk colorectal surgery laparotomy wounds. Color Dis. (2019) 21:110–8. doi: 10.1111/codi.14350, 30047611 PMC7380040

[11] Gombert A Babilon M Barbati ME Keszei A von Trotha KT Jalaie H . Closed incision negative pressure therapy reduces surgical site infections in vascular surgery: a prospective randomised trial (AIMS trial). Eur J Vasc Endovasc Surg. (2018) 56:442–8. doi: 10.1016/j.ejvs.2018.05.018, 29970335

[12] Kwaan MR Weight CJ Carda SJ Mills-Hokanson A Wood E Rivard-Hunt C . Abdominal closure protocol in colorectal, gynecologic oncology, and urology procedures: a randomized quality improvement trial. Am J Surg. (2016) 211:1077–83. doi: 10.1016/j.amjsurg.2015.10.032, 26850135

[13] Mohan N Gnanasekar D Tk S Ignatious A. Prevalence and risk factors of surgical site infections in a teaching medical College in the Trichy District of India. Cureus. (2023) 15:e39465. doi: 10.7759/cureus.39465, 37362535 PMC10290230

[14] Zeng JK Sun XJ Sun ZY Guan J Han C Zhao X . Negative pressure wound therapy versus closed suction irrigation system in the treatment of deep surgical site infection after lumbar surgery. World Neurosurg. (2019) 127:E389–e395. doi: 10.1016/j.wneu.2019.03.130, 30905647

[15] Norman G Shi CH Goh EL Murphy EMA Reid A Chiverton L . Negative pressure wound therapy for surgical wounds healing by primary closure. Cochrane Db Syst Rev. (2022) 4:CD009261. doi: 10.1002/14651858.CD009261.pub4 PMC9040710 35471497

[16] Li HZ Xu XH Wang DW Lin YM Lin N Lu HD. Negative pressure wound therapy for surgical site infections: a systematic review and meta-analysis of randomized controlled trials. Clin Microbiol Infec. (2019) 25:1328–38. doi: 10.1016/j.cmi.2019.06.005, 31220604

[17] Brennfleck FW Linsenmeier L Junger HHG Schmidt KM Werner JM Woehl D . Negative pressure wound therapy (NPWT) on closed incisions to prevent surgical site infection in high-risk patients in hepatopancreatobiliary surgery: study protocol for a randomized controlled trial-the NP-SSI trial. Trials. (2020) 21:918. doi: 10.1186/s13063-020-04831-z, 33168081 PMC7654160

[18] Andrianello S Landoni L Bortolato C Iudici L Tuveri M Pea A . Negative pressure wound therapy for prevention of surgical site infection in patients at high risk after clean-contaminated major pancreatic resections: a single-center, phase 3, randomized clinical trial. Surgery. (2021) 169:1069–75. doi: 10.1016/j.surg.2020.10.029, 33257037

[19] Morykwas MJ Argenta LC Shelton-Brown EI McGuirt W. Vacuum-assisted closure: a new method for wound control and treatment: animal studies and basic foundation. Ann Plast Surg. (1997) 38:553–62. doi: 10.1097/00000637-199706000-00001, 9188970

[20] van Dam MA Strietman M van Eps RGS Wever JJ Veger HTC. Clinical relevance of closed-incision negative pressure therapy (ciNPT) for SSI-risk reduction in vascular surgery through a groin incision. Ann Vasc Surg. (2022) 78:93–102. doi: 10.1016/j.avsg.2021.06.035, 34537352

[21] Horch RE. Incisional negative pressure wound therapy for high-risk wounds. J Wound Care. (2015) 24:21–8. doi: 10.12968/jowc.2015.24.Sup4b.21, 25853645

[22] Kim JH Lee DH. Are high-risk patient and revision arthroplasty effective indications for closed-incisional negative-pressure wound therapy after total hip or knee arthroplasty? A systematic review and meta-analysis. Int Wound J. (2020) 17:1310–22. doi: 10.1111/iwj.13393, 32406175 PMC7948573

[23] Abatangelo S Saporiti E Giatsidis G. Closed incision negative-pressure therapy (ciNPT) reduces minor local complications in post-bariatric abdominoplasty body contouring: a retrospective case-control series. Obes Surg. (2018) 28:2096–104. doi: 10.1007/s11695-018-3279-8, 29730777

[24] Willy C Agarwal A Andersen CA Santis G Gabriel A Grauhan O . Closed incision negative pressure therapy: international multidisciplinary consensus recommendations. Int Wound J. (2017) 14:385–98. doi: 10.1111/iwj.12612, 27170231 PMC7949983

[25] Redfern RE Cameron-Ruetz C O'Drobinak SK Chen JT Beer KJ. Closed incision negative pressure therapy effects on postoperative infection and surgical site complication after Total hip and knee arthroplasty. J Arthroplast. (2017) 32:3333–9. doi: 10.1016/j.arth.2017.06.019, 28705547

[26] Ge D. The safety of negative-pressure wound therapy on surgical wounds: an updated Meta-analysis of 17 randomized controlled trials. Adv Skin Wound Care. (2018) 31:421–8. doi: 10.1097/01.ASW.0000542530.71686.5c, 30134278

[27] Seidelman JL Mantyh CR Anderson DJ. Surgical site infection prevention a review. Jama-J Am Med Assoc. (2023) 329:244–52. doi: 10.1001/jama.2022.24075, 36648463

[28] Meyer J Roos E Davies RJ Buchs NC Ris F Toso C. Does prophylactic negative-pressure wound therapy prevent surgical site infection after laparotomy? A systematic review and Meta-analysis of randomized controlled trials. World J Surg. (2023) 47:1464–74. doi: 10.1007/s00268-023-06908-7, 36658232 PMC10156868

[29] Ma Y Shen K Zhou X Zhang P Lu Z. A novel mini-open transforaminal lumbar interbody fusion for lumbar degenerative diseases: technical note and preliminary results. J Orthop Surg Res. (2023) 18:517. doi: 10.1186/s13018-023-04018-7, 37475005 PMC10360288

[30] Soliman AM Barreda DR. Acute inflammation in tissue healing. Int J Mol Sci. (2023) 24:641. doi: 10.3390/ijms24010641, 36614083 PMC9820461

[31] Martin P Pardo-Pastor C Jenkins RG Rosenblatt J. Imperfect wound healing sets the stage for chronic diseases. Science. (2024) 386:eadp2974. doi: 10.1126/science.adp2974, 39636982 PMC7617408

